# An In-Vitro Evaluation of Strength, Hardness, and Color Stability of Heat-Polymerized and 3D-Printed Denture Base Polymers After Aging

**DOI:** 10.3390/polym17030288

**Published:** 2025-01-23

**Authors:** Abdulrahman Al-Ameri, Othman Y. Alothman, Omar Alsadon, Durgesh Bangalore

**Affiliations:** 1 Department of Chemical Engineering, College of Engineering, King Saud University, Riyadh 12372, Saudi Arabia; abdu.m.a9311@gmail.com (A.A.-A.); othman@ksu.edu.sa (O.Y.A.); 2 Dental Health Department, College of Applied Medical Sciences, King Saud University, Riyadh 12372, Saudi Arabia; oalsadon@ksu.edu.sa

**Keywords:** 3D-printing, color, denture base resins, mechanical properties, optical properties, flexural strength

## Abstract

This study evaluated the strength, hardness, and color stability of 3D-printed denture base resins and compared the outcome with conventional heat-cured denture base resins after aging by thermocycling. A total of 72 specimens from conventional and 3D-printed materials were fabricated in different shapes and dimensions based on the mechanical and color tests performed. The specimens were divided into five groups: flexural, tensile, and compressive strengths (n = 20), hardness, and color stability (n = 6). In all these groups, half of the specimens were stored in a distilled water bath at 37 °C for 24 h, and the remaining half of the specimens were subjected to aging by thermocycling. The 3D-printed specimens demonstrated the highest means of tensile strength (32.20 ± 3.8 MPa), compressive strength (106.31 ± 4.07 MPa), and Vickers hardness number (24.51 ± 0.36), and the lowest means of flexural strength (54.29 ± 13.17 MPa) and color difference (ΔE = 2.18 ± 1.09). Conventional heat-cured specimens demonstrated the highest means of flexural strength (59.96 ± 8.39 MPa) and color difference (ΔE = 4.74 ± 2.37) and the lowest means of tensile strength (32.17 ± 9.06 MPa), compressive strength (46.05 ± 4.98 MPa), and Vickers hardness number (10.42 ± 1.05). Aging significantly reduced the flexural strength (−27%), tensile strength (−44%), and hardness (−7%) of 3D-printed resins in contrast to the conventional resin’s compressive strength (−15%) and color stability (*p* < 0.05). The 3D-printed resin had comparable flexural and tensile strength and significantly superior compressive strength, hardness, and color stability compared with conventional resins. Aging significantly and negatively affected the flexural strength, tensile strength, and hardness of 3D-printed resin.

## 1. Introduction

Polymethyl methacrylate (PMMA) is a thermoplastic amorphous polymer synthesized from methacrylate monomers [1]. Due to its significantly high hemo- and biocompatibility, optical characteristics, and ease of handling, PMMA is used in several medical and dental applications [2]. Consequently, PMMA is a viable option for producing cranioplasty implants, blood pumps, dialyzers, intraocular implants, hard contact lenses, and oral appliances [3,4].

PMMA has historically dominated denture fabrication in dentistry and is the gold standard material for completely edentulous patients [5]. Conventional PMMA resins have the following advantages: stability, affordability, satisfactory aesthetics, and ease of handling [5,6,7]. However, the use of PMMA is limited by its dimensional instability, surface voids, residual monomer concentration, fracture vulnerability, and low resistance to wear in human saliva, as well as allergic reactions and the gradual deterioration of its mechanical qualities [6,7,8]. Traditionally, PMMA has been commonly processed by compression and injection molding methods [9,10]. However, these processing methods have inherent drawbacks, such as multiple appointments, bulky prostheses for maxillofacial patients, greater vulnerability to bacterial colonization, and visible polymerization shrinkage [9,11].

The fabrication of dentures has undergone significant changes in recent years owing to the development of newer materials and fabrication techniques such as computer-aided design and computer-aided manufacturing (CAD/CAM) methods [6,12,13]. The use of CAD/CAM techniques in denture fabrication has garnered much interest, leading to improvements in both design and production, with the promise of faster and better results [14]. Digital dentures have demonstrated reasonable cost-effectiveness, good patient-related outcomes, and encouraging short-term clinical performance [8]. Digital denture production initially emerged as a subtractive process undertaken by the milling of prepolymerized multilayered PMMA discs [15]. The main drawback of milling is that a significant amount of the blank is wasted and left unusable during the process [8]. Furthermore, the contour of the prosthesis depends on the size of the cutting tools, which is one of the constraints of the milling process. The accuracy of the internal fit or the marginal characteristics will be deteriorated if the cutting tool’s diameter is greater than the diameter of some components [16].

Additive manufacturing (AM), sometimes referred to as rapid prototyping (RP) or three-dimensional (3D) printing, includes methods that create objects by layering photopolymerizable resin using liquid crystal screens or laser and visible light to cure the resin [8,17,18]. Despite being relatively new, 3D printing has demonstrated promise in a variety of disciplines, including engineering and medicine, especially dentistry [8]. The new AM technology is revolutionizing the clinical and laboratory procedures for fabricating dentures [8].

Compared with milling technology, 3D printing has the advantage of being able to create more complex material shapes because it is not constrained by milling bur accessibility [19]. Additionally, 3D printing machines are less expensive than milling machines, and material waste can be decreased [20,21]. Patients have expressed excellent satisfaction levels with 3D-printed dentures [22], which are more precise and fit better than conventional dentures [23]. However, 3D printing has certain drawbacks such as high cost of materials and processing, allergic stomatitis, time-consuming post-processing curing, and increased roughness due to layer-by-layer printing [24,25].

The clinical performance of dental materials is greatly influenced by their mechanical characteristics, which are closely associated with both composition and processing. Complete dentures, which are used to restore both function and appearance in edentulous patients, are susceptible to fractures and fatigue due to a variety of intricate masticatory forces in the intraoral environment, including flexural, tensile, and compressive [26,27,28]. The hardness of a dental material is important in order to endure the abrasive action of mastication and preserve visual appeal [29]. Another important property is denture color stability, i.e., the ability of denture materials to maintain their original color and appearance over time [30].

Dentures and other dental appliances are constantly exposed to temperature changes occurring due to food and beverages. The characteristics of appliances may be negatively impacted by these temperature fluctuations, especially if they are repeated frequently. Therefore, testing the efficacy of denture base materials under conditions similar to those in the intraoral environment is crucial. The oral cavity’s temperature fluctuates 20–50 times daily, with an average of 10,000 times annually [29].

Three-dimensional printing technology promises to revolutionize the prosthodontics specialty, primarily in the fabrication of denture prostheses, but its full potential has yet to be understood. This is due to the paucity of studies and research that contrast the properties of materials fabricated using conventional techniques with those obtained by 3D printing. There are quite a few existing studies evaluating the mechanical properties and surface properties [30,31,32,33,34,35,36], but none of them include a comprehensive analysis, and additionally the outcomes of these studies are conflicting. Thus, it was recommended that a further thorough analysis be conducted in order to clearly understand the properties of 3D-printed denture base resins.

Consequently, this study aimed to compare the mechanical properties and color stability of 3D-printed denture base resins and compare the outcome with conventional heat-cured denture base resins after aging by thermocycling. The comparison was undertaken through mechanical tests (flexural strength, tensile strength, compressive strengths, and hardness) and color stability. It was hypothesized that there would be no significant difference in the mechanical and optical properties of 3D-printed heat-cured and conventional heat-cured denture base resins.

## 2. Materials and Methods

In this study, a heat-cured and a 3D-printed denture base resin material were evaluated (Table 1).

### 2.1. Sample Preparation

**Figure 1:**
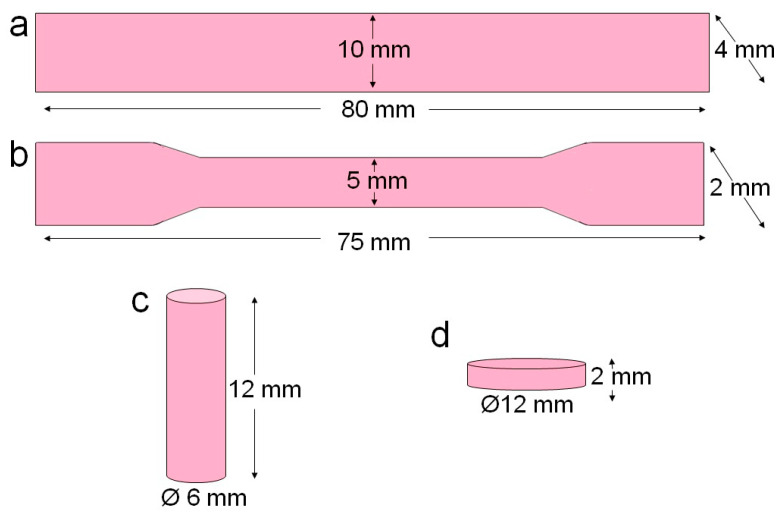
Specimen shapes and dimensions used in this study. (**a**) flexural strength, (**b**) Tensile strength, (**c**) compressive strength and (**d**) hardness and color stability.

Seventy-two specimens from each resin material (in total 142 specimens) were fabricated with different shapes and dimensions based on the type of tests performed (Figure 1). All specimens were prepared as per the ISO specifications, which are mentioned under each test. The specimens were divided into five test groups: flexural (n = 20), tensile (n = 20), and compressive strengths (n = 20); hardness (n = 3); and color stability (n = 3). In all these groups, half of the specimens were stored in a distilled water bath at 37 °C for 24 h, and the remaining half of the specimens were subjected to aging by thermocycling. All the specimens were prepared using International Organization for Standardization (ISO) guidelines.

For the fabrication of heat-cured acrylic resin, molten wax was initially poured into a silicone mold and allowed to cool until it obtained the required shape. These wax samples were then processed using the flasking technique to produce conventional heat-cured acrylic samples. The conventional PMMA resin was mixed (21 g powder: 10 mL liquid) according to the manufacturer’s instructions and packed into the mold space when the resin mix was at the dough stage. The denture flask was clamped in a bench press with slow pressure to ensure uniform material flow within the mold. The specimen was polymerized in a water bath curing system at an initial temperature of 25 °C, steadily increasing to 70 °C. The flask remained in the water bath for 2 h and was later removed and allowed to cool at room temperature [37]. Finally, the specimens were removed from the flask, and excess resin was trimmed.

For the fabrication of 3D-printed specimens, virtual specimens were designed using CAD software (Exocad GmbH, Darmstadt, Germany) and saved as a standard tessellation language (.STL) file. The .STL file was imported via the Preform print software, and the specimens were printed using an SLA 3D printer (Formlabs 2, Formlabs Inc., Somerville, MA, USA) at 0° and a 50 μm z-axis layer height. After printing, the specimens were cleaned in 99% isopropyl alcohol (Form Wash, Formlabs Inc., Somerville, MA, USA) and then allowed to dry. The support structures were removed, and the specimens were placed in a light polymerization unit for 60 min to allow further polymerization of the printed specimens [6]. All the specimens from both materials were polished as per a previous study [6,38].

The dimensions of the specimens were confirmed using a digital caliper. Next, 50% of the specimens from both material groups were placed in distilled water at 37 °C for 24 h. The remaining 50% of the specimens were subjected to thermocycling (5000 cycles, 5–55 °C, 30 s dwell time, and 10 s transfer time; SD Mechatronik GmbH, Feldkirchen-Westerham, Germany) [6,29]. A set of 5000 cycles equaled six months of oral usage [6,29]. The specimens which had been either stored in distilled water (baseline) or thermocycled were subjected to mechanical and color tests as detailed below. Table 2 presents a description of the different tests and specimens used in this study.

### 2.2. Specimen Testing

#### 2.2.1. Flexural Strength

**Figure 2:**
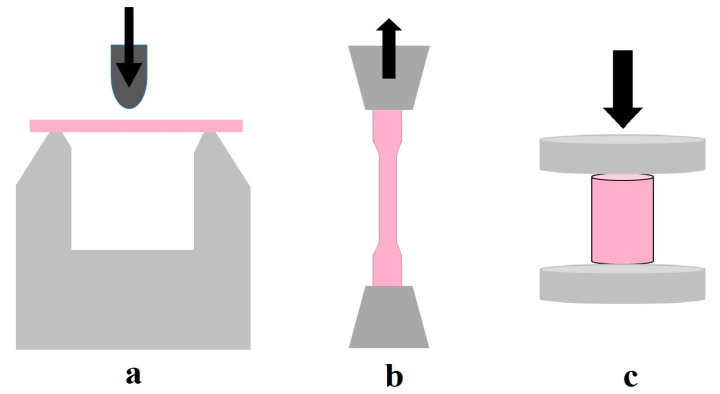
Schematic illustration of strength test set-up. (**a**) Flexural, (**b**) Tensile, (**c**) Compressive.

Twenty rectangular samples (80 mm length × 10 mm width × 4 mm thickness) were prepared from each resin material for flexural strength testing according to the ISO 178:2019 guidelines (Figure 1a). The flexural strength test was performed in a three-point bending test set-up at room temperature in a universal testing machine (H100KS, Tinius Olsen, Horsham, PA, USA). The specimen was placed horizontally on a three-point flexure apparatus at a distance of 64 mm between the two supports. A vertical load was applied at the center of the specimen with a crosshead speed of 0.5 mm/min until the specimen fractured (Figure 2a). The fracture load was recorded, and the flexural strength was determined using Equation (1):

(1) $$S=\frac{3PL}{2bd^{2}}(MPa)$$

where P is the load at the fracture point on the load-deflection curve (N), L is the distance between the supports (64 mm), b is the specimen width (10 mm), and d is the specimen thickness (4 mm).

The elastic modulus (Y) was determined based on the flexural strength test results using Equation (2):

(2) $$Y=\frac{FL^{3}}{4bh^{3}d}(GPa)$$

where E is the elastic modulus (GPa), F is the load (N) at a chosen point (P) on the tension/deformation curve (elastic deformation), L is the distance between the two supports, b is the specimen width, h is the specimen thickness, and d is the deflection at the point (P).

#### 2.2.2. Tensile Strength

Twenty dumbbell-shaped specimens were prepared from each resin material (75 mm length × 5 mm width at the mid region × 2 mm thickness) according to the ISO 527-2:2012 guidelines (Figure 1b). The flexural strength test was performed at room temperature in a universal testing machine (H100KS, Tinius Olsen, Horsham, PA, USA). The specimen was initially mounted on the upper assembly, allowing it to hang freely and retain alignment during testing. The lower assembly engaged the other end of the specimen. A tensile load at 0.5 mm/min (P) in the opposite direction to the specimen was applied (Figure 2b), and the maximum load that caused fracture was recorded. Tensile strength was determined using Equation (3):

(3) $$\sigma =\frac{Load}{Area}=\frac{P}{a}$$

#### 2.2.3. Compressive Strength

Twenty cylindrical specimens were prepared from each resin material (Ø 6 mm × 12 mm length) (Figure 1c) according to the ISO 5883:2002 guidelines for compressive strength testing. The test was performed at room temperature in a universal testing machine (H100KS, Tinius Olsen, Horsham, PA, USA) with a test speed of 0.5 mm/min (P) (Figure 2c). The behavior of the materials under crushing load was determined using Equation (4):

(4) $$CS=\frac{Load}{Area}=\frac{P}{a}$$

Figure 2 presents the schematic presentation of the strength test set-up.

#### 2.2.4. Hardness

For hardness testing, six round specimens were prepared from each resin material (Ø 12 mm × 2 mm thickness) (Figure 1d). The microhardness of the specimens was determined using the Vickers micro-hardness tester (INNOVATEST Europe BV, Borgharenweg, The Netherlands). A diamond pyramid indenter with a square base on the opposite side at an angle of 136° was used to indent the material surface with a force of 1 kgf. Each specimen was subjected to three indentations, 0.5 mm apart and with 15 s dwell time, and the hardness number was identified with a minimal margin of error. The test conditions were in accordance with ISO 6507-1:2023 specifications [44,45]. The diamond-shaped indent on the material surface was observed under a microscope, and its diagonal was measured to determine the Vickers hardness (HV) using Equation (5):

(5) $$HV=1854\times 10^{-3}\frac{F}{d^{2}}\;$$

where F is the load in kgf, d is the arithmetic means of the two diagonals (mm), and HV is the Vickers hardness.

#### 2.2.5. Color Stability

Color stability of the specimens was in accordance with ISO 28642:2016 specifications. Six round specimens were prepared from each resin material (Ø 12 mm × 2 mm thickness) (Figure 1d) to determine the color stability using a Lab Scan XE spectrophotometer (Hunter Associates Laboratory, Inc., Reston, VA, USA). The color reading against a white background was determined at baseline and after thermocycling at three random locations at its center for each specimen. The average of all measurements was recorded, and the CIELab uniform color scale was used to determine the mean color change (∆E_ab_) from baseline to thermocycling using Equation (6):

(6) ∆Eab=[∆L*2+∆a*2+∆b*2]12

The value of L* at 0 is indicated as black, and at 100 it is indicated as white; the value of a*, if positive, is indicated as red, and if negative, is indicated as green. If positive, the value of b* is indicated as yellow; if negative, it is blue. A ΔE_ab_ ≤ 3.3 was considered a clinically acceptable reference value [46].

#### 2.2.6. Fourier-Transform Infrared Spectroscopy (FTIR) Analysis

In comparing the material composition between the conventional and 3D-printed resins, the infrared performance of the materials was determined using FT-IR spectrophotometer (Perkin Elmer, Buckinghamshire, UK). As each material absorbs infrared light in a particular manner, waveform data with each peak corresponding to a different functional group was obtained in the 400 and 4000 cm^−1^ wavenumber region, 4 cm^−1^ resolutions, and an average of 20 scans at room temperature. The materials were tested using the PMMA reference peaks following a previous study [47].

The data collected from tests of flexural strength, elastic modulus, tensile strength, compressive strength, hardness, and color stability were tabulated and analyzed using a statistical package for the social sciences software (V.22, IBM^®^, SPSS^®^, Chicago, IL, USA). A Shapiro-Wilk test revealed normal distribution of the data. Mean and standard deviations were computed using one-way ANOVA for each test. The analyzed data at baseline and after thermocycling were compared using Tukey’s multiple pairwise comparisons test to determine significant differences between them (*p* ≤ 0.05).

## 3. Results

### 3.1. Flexural Strength

**Figure 3:**
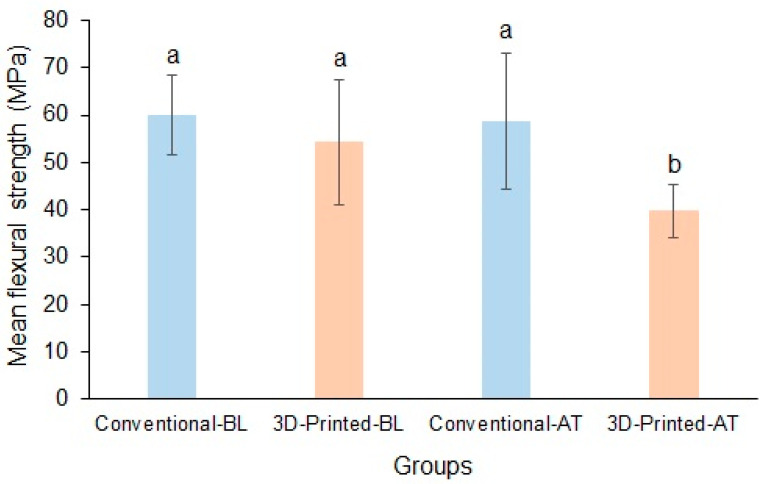
Mean flexural strength of conventional and 3D-printed resins at baseline and after thermocycling (Baseline, BL; Thermocycled, AT). Materials with the same lowercase letters are not significantly different.

The mean baseline flexural strengths of the conventional and 3D-printed resins were 59.96 ± 8.39 MPa and 54.29 ± 13.17 MPa, respectively. Following thermocycling, the mean flexural strengths of the conventional and 3D-printed resins were 58.64 ± 14.3 MPa and 39.79 ± 5.6 MPa, respectively, and the difference between the materials was significant (*p* < 0.05). The flexural strength of the 3D-printed resin decreased significantly after thermocycling (Figure 3).

#### Elastic Modulus

**Figure 4:**
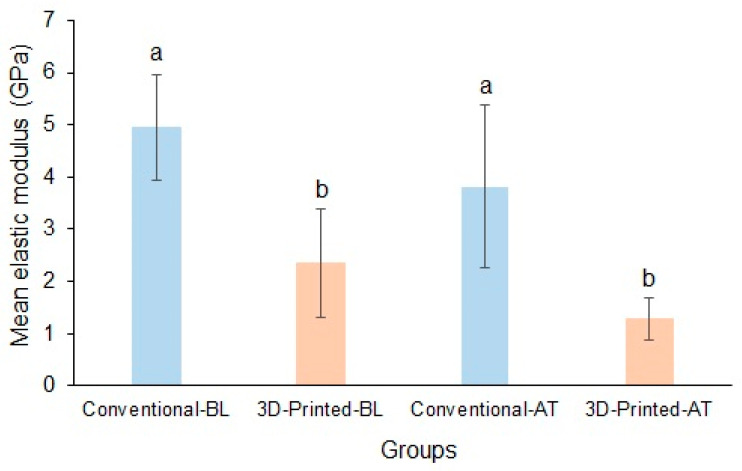
Mean elastic modulus of conventional and 3D-printed resins at baseline and after thermocycling (Baseline, BL; Thermocycled, AT). Materials with the same lowercase letters are not significantly different.

The mean baseline elastic moduli of the conventional and 3D-printed resins were 4.95 ± 1.00 GPa and 2.36 ± 1.04 GPa, respectively. Following thermocycling, the mean flexural moduli of the conventional and 3D-printed resins were 3.82 ± 1.57 GPa and 1.29 ± 0.4 GPa, respectively. Both the baseline and thermocycled elastic moduli were significantly different between the materials (*p* < 0.05). However, thermocycling did not affect the elastic modulus in both materials (Figure 4).

### 3.2. Tensile Strength

**Figure 5:**
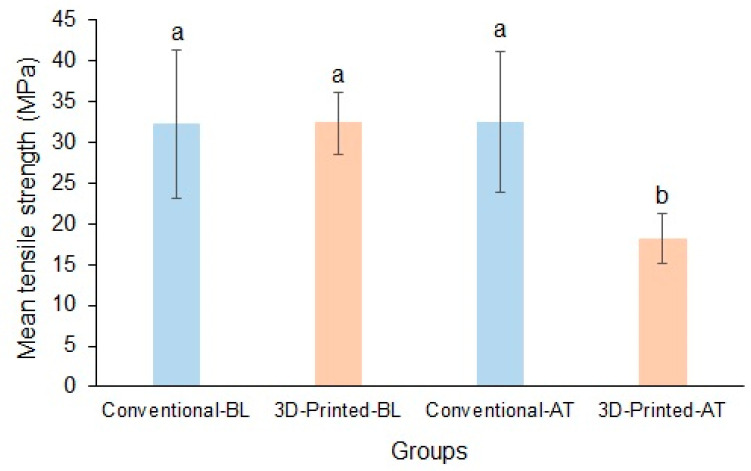
Mean tensile strength of conventional and 3D-printed resins at baseline and after thermocycling (Baseline, BL; Thermocycled, AT). Materials with the same lowercase letters are not significantly different.

The mean baseline tensile strengths of the conventional and 3D-printed resins were 32.17 ± 9.06 MPa and 32.30 ± 3.86 MPa, respectively, and the difference between the materials was insignificant. Following thermocycling, the mean tensile strengths of the conventional and 3D-printed resins were 32.39 ± 8.64 MPa and 18.10 ± 3.04 MPa, respectively, and the difference between the materials was significant (*p* < 0.05). The results also showed that the tensile strength of conventional resins was resistant to thermal changes, whereas that of 3D-printed resins was not (Figure 5).

### 3.3. Compressive Strength

**Figure 6:**
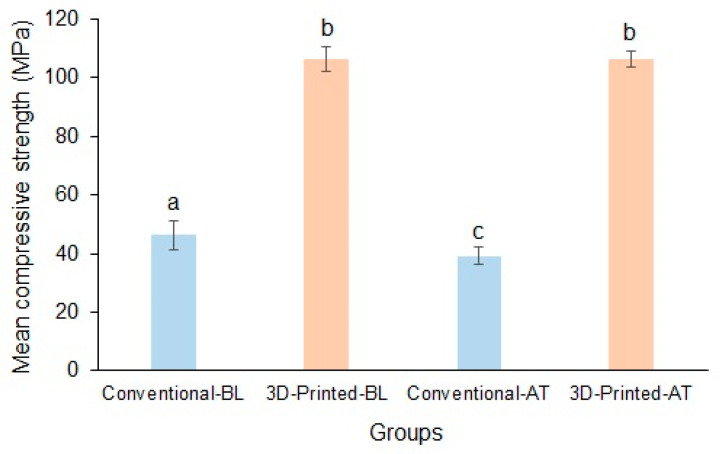
Mean compressive strength of conventional and 3D-printed resins at baseline and after thermocycling (Baseline, BL; Thermocycled, AT). Materials with the same lowercase letters are not significantly different.

The mean baseline compressive strengths of the conventional and 3D-printed resins were 46.05 ± 4.98 MPa and 106.31 ± 4.07 MPa, respectively, and the difference between the materials was significant. Following thermocycling, the mean compressive strengths of the conventional and 3D-printed resins were 39.21 ± 3.04 MPa and 106.28 ± 2.61 MPa, respectively, and the difference between the materials was significant (*p* < 0.05). The compressive strength of the 3D-printed resin was resistant to thermal changes compared with that of the conventional resin (Figure 6).

### 3.4. Hardness

**Figure 7:**
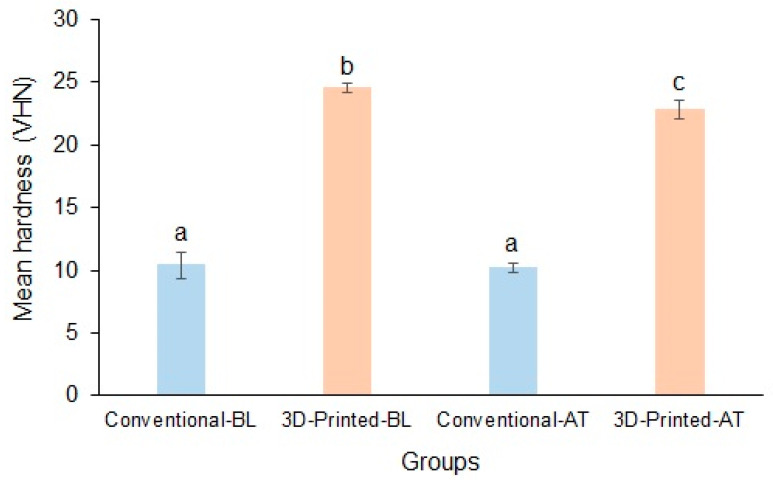
Mean hardness of conventional and 3D-printed resins at baseline and after thermocycling (Baseline, BL; Thermocycled, AT). Materials with the same lowercase letters are not significantly different.

The mean baseline hardnesses of the conventional and 3D-printed resins were 10.42 ± 1.05 VHN and 24.51 ± 0.36 VHN, respectively, and the difference in hardness between the materials was significant. Following thermocycling, the mean hardnesses of the conventional and 3D-printed resins were 10.23 ± 0.39 VHN and 22.82 ± 0.69 VHN, respectively, and the difference between the materials was significant (*p* < 0.05). The hardness of the conventional resin was resistant to thermal changes compared with that of the 3D-printed resin (Figure 7).

### 3.5. Color Difference

The CIE lab uniform color scale changed with the type of material and was also affected by thermocycling (Table 3). A significant difference was observed between the material types at baseline and after thermocycling for L, a, and b values (*p* < 0.05).

**Figure 8:**
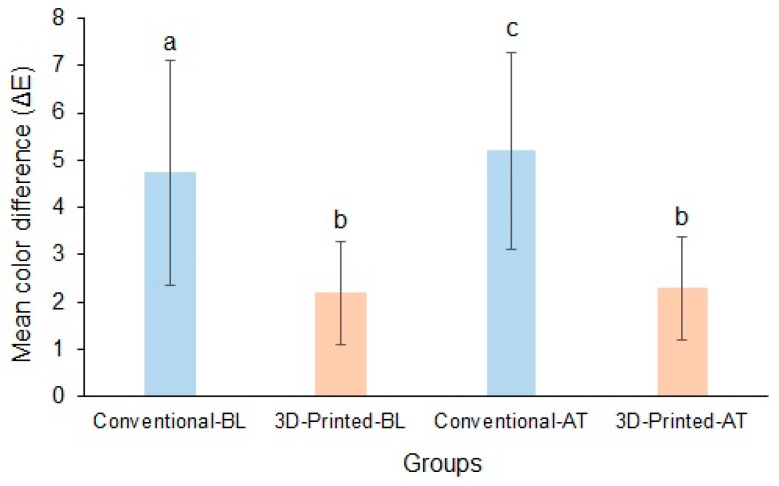
Mean ΔE of conventional and 3D-printed resins at baseline and after thermocycling (Baseline, BL; Thermocycled, AT). Materials with the same lowercase letters are not significantly different.

The mean baseline ΔE values of the conventional and 3D-printed resins were ΔE_ab_ = 4.74 ± 2.37 and ΔE = 2.18 ± 1.09, respectively, and the difference in ΔE value was significant between the materials. Following thermocycling, the mean ΔE values of the conventional resin and 3D-printed resin were ΔE_ab_ = 5.2 ± 2.07 and ΔE_ab_ = 2.3 ± 1.09, respectively. The material type significantly affected ΔE_ab_ at baseline and after thermocycling. The ΔE_ab_ of the conventional resin was significantly affected by thermal changes compared with that of the 3D-printed resin (*p* < 0.05). The mean ΔE value of the conventional resin was above the clinically acceptable values (ΔE_ab_ ≥ 3.3) for both measurements, in contrast to the 3D-printed resin, which exhibited ΔE_ab_ values below the clinically acceptable range (ΔE_ab_ ≤ 3.3) (Figure 8).

### 3.6. FTIR Analysis

**Figure 9:**
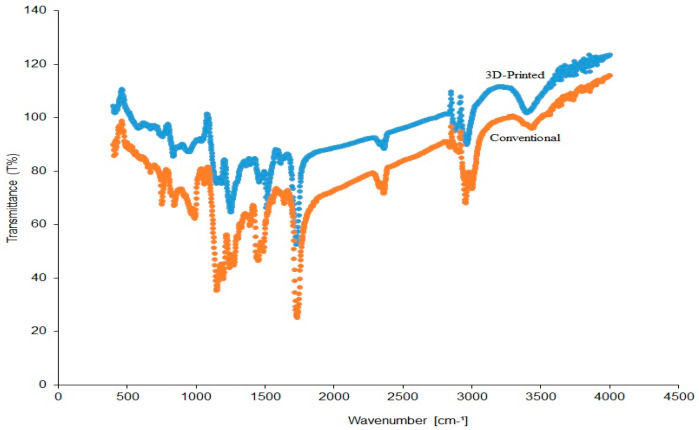
FTIR waveforms of the conventional and 3D-printed resins.

Table 4 presents the diagnostic IR absorption of the study materials based on the reference PMMA. There were no noticeable variations in the wavenumber concerning the functional groups of both materials. The FTIR peaks showed similar peak forms for both materials (Figure 9), which infers that both materials were primarily composed of monomers based on methacrylic esters.

## 4. Discussion

This study evaluated and compared the effect of aging by thermocycling on the mechanical properties and color stability of 3D-printed and conventional heat-cured denture base resins. The flexural strength, tensile strength, compressive strength, hardness, and color stability of the resins were analyzed. It was hypothesized that there would be no significant difference in the mechanical properties and color stability of 3D-printed and conventional heat-cured denture base resins. The study’s comparison elucidated that the 3D-printed resin had comparable flexural and tensile strength and significantly superior compressive strength, hardness and color stability compared with the conventional resin. Thus, the outcome of the study recommends a null hypothesis rejection.

Flexural strength and elastic modulus: By analyzing its mechanical characteristics, one may predict a material’s resistance to the stresses induced by mastication [26]. One of the main aspects of PMMA-based dentures is the flexural characteristics, which reflect the various stresses exerted on the denture during mastication and provide a sense of material rigidity [26]. Flexural strength is defined as the maximum flexing stress a dental material can withstand before it fractures [48]. The material used to fabricate dentures must have a high flexural strength because a denture base can fracture or fail clinically for several reasons, such as mastication fatigue, or extra-orally when not being worn due to unanticipated incidents. Dentures with low flexural strength are prone to cracks and fracture as they cannot tolerate the constant stresses caused by mastication [26].

In the current study, both resins had comparable flexural strength at baseline, which is consistent with the findings of the previous studies [34,35]. Fiore et al. [34] and Sonam et al. [35] demonstrated no significant differences between the flexural strength values of the conventional and 3D-printed groups. Based on the outcomes of their studies, the authors concluded that the 3D-printed materials are a viable option for fabricating denture bases. The current study’s outcome contradicts with the findings by Ataei et al. [49], where the authors demonstrated significantly low flexural strength compared with heat-cured resin. Furthermore, the current study also contradicts with the findings of Temizci and Bozoğulları [45], where the authors found 3D-printed resins to have significantly high flexural strength compared with heat-cured conventional resins. In the current study, after thermocycling, the printed resin showed a significant decrease in flexural strength. Conventional heat-cured acrylics fabricated via conventional methods were more resistant to temperature variations. The aging of 3D-printed resins results in decreased flexural strength due to factors such as water absorption, variations in the printed layers, and material composition [50]. A similar such outcome has been demonstrated by Gad et al. [29] in their recent study evaluating the effect of thermocycling on 3D-printed denture base resin. Conversely, Reddy et al. [51] showed that the flexural strength of a printed material improved after thermocycling. The authors also confirmed that thermal stress had no significant effect on the flexural strength of both tested materials which also contradicts the findings of our study with regard to 3D-printed resins. In a study by Alaseef et al. [52], it was found that 3D-printed specimens with a minimum thickness of 2 mm yielded acceptable flexural strength compared with other thicknesses (1.5 mm, 2.5 mm and 3.3 mm).

The denture base material should have a high elastic modulus or be rigid enough to avoid permanent deformation from the stresses exerted during biting and mastication. The elastic modulus, reflecting the rigidity of the denture resins, demonstrated that the conventional resins had the highest rigidity and 3D-printed resins had lowest, both at baseline and after aging. Furthermore, the rigidity of both materials was significantly affected by thermal stresses. AlQarni et al. [53] and Zeidan et al. [54], evaluating the flexural modulus, also demonstrated the low elastic modulus of printed resins compared with conventional resins. The flexural modulus of both materials at baseline was in the range of 3.82–4.95 GPa, but printed resin demonstrated a significant decrease in elastic modulus (1.29 GPa) after thermocycling. It has been determined that residual monomers in 3D-printed specimens are implicated in the poor performance of 3D-printed resins, which leads to the formation of voids, weaker double bonds, and poor adhesion between succeeding layers [53]. Furthermore, residual monomer in the 3D-printed resins also acts as a plasticizer, which also reduces the elastic modulus [54].

Tensile strength: The measurement of the highest stretching or elongation force that can be applied continually across a material’s cross-section is known as its tensile strength. High tensile strength is one of the essential mechanical characteristics of a denture base resin, crucial for the material to function as intended [55]. Similar to flexural strength, the baseline tensile strength was comparable between the tested denture resins. However, the 3D-printed resin demonstrated significantly reduced tensile strength after thermocycling. One possible explanation of the aged printed material’s diminished mechanical properties is the thermal stressing that affects the layering interface. Higher water temperatures increase water sorption, which causes resin to swell and disengage from printed layers. This, in turn, may have an impact on the printed resin’s strength [56]. Conventional PMMA has more stable flexural and tensile strengths when exposed to thermocycling as it is thermally resistant and can withstand temperatures up to 450 °C, after which the material begin to depolymerize [30]. Furthermore, variations in composition of the resin material, printed layer width, appropriate printer and software, printing orientation, and post-print curing process can affect the properties of the printed product [57]. This could also be the reason for different outcomes among existing studies. The poor mechanical properties of dentures fabricated by 3D printing can also be explained by the consolidation of the reactivity of 3D-printed resin monomers and the curing conditions, which result in a lower degree of double-bond conversion than conventional acrylic resins [30].

Another important consideration during 3D printing of denture base resin is the orientation. In the current study, the 3D-printed specimens were printed at 0° orientations and this could have accounted for the low strength values. Altarazi et al. [58] demonstrated that a 90° orientation can provide higher mechanical and physical properties.

Compressive strength: Most mastication forces are compressive, and a compressive strength test mimics the load applied to materials used in dental practice [59]. As a result, compressive strength is a relevant characteristic for comparing denture resins. Although they are referred to as compression strength equations, the mathematical equations created for the tensile force are also used to calculate the compression strength. Because the polymer material can handle a pulling load differently from a columnar load, the compression strength can differ significantly from the tensile strength [60]. In contrast to flexural and tensile strength, the compressive strength of the printed material was significantly higher than that of the conventional resin. Furthermore, the conventional resin was significantly affected by thermal stresses in contrast to the printed resin. Among the previous studies, only one study by Arora et al. [36] evaluated the compressive strength of 3D-printed resin. The authors found that the compressive strength of 3D-printed resins (86.05 MPa) was comparable with one injection-molded PMMA (Polyan IC; 95.11 MPa) but significantly higher than the other injection-molded PMMA (SR Ivocap; 68.55 MPa). Printing orientation is reported to have a significant impact on the compressive strength of resin materials. Alharbi et al. [61]. demonstrated that the specimens printed horizontally, with the layers aligned parallel to the direction of load, had a substantially reduced compressive strength compared with the vertically printed specimens. The intersection of the layers was in the path of the load application in the horizontally printed specimens.

Hardness: This is another essential mechanical characteristic which indicates how resistant a material is to plastic deformation. Therefore, a denture resin’s hardness suggests the possibility that the polymer matrix may deteriorate. Consequently, the matrix deteriorates with decreasing hardness, raising the possibility of material fracture and the risk of discoloration and microbial retention. Thus, the clinical longevity of the denture base is declined [62]. The minimum Vickers hardness number requirement for denture-based polymers per ISO specifications is at least 16.00 VHN [63]. The results of the current study showed that the 3D-printed resin had a significantly higher VHN that was above the optimal limit (24 VHN) compared with the conventional heat-cured resin (10 VHN). Following aging, the VHN of the printed material decreased significantly but was well within the optimal limit in contrast to the non-significant decrease in the conventional resin. Similar to the current study’s outcomes, Digholkar et al. [64] evaluated the VHN of interim crown and bridge materials and found significantly a higher VHN with 3D-printed resins compared with heat-cured and milled PMMA resins. This difference in VHN was attributed to the different internal compositions of the materials. Conversely, this study’s outcome was in disagreement with previous studies demonstrating a significantly high VHN in heat-cured denture resins compared with 3D-printed denture resins [12,36,65]. Al-Dwairi et al. [12], Prpic et al. [65], and Arora et al. [36] showed that the hardness of a 3D-printed resin was comparatively less than that of conventional heat-cured PMMA. The possibility of this variation was the material’s density, which varies and may be related to the filler content in the case of 3D-printed resins. However, it is essential to exercise caution when interpreting the results between studies because of the variations among commercial brands of resins, compositions and structures, pre- and post-processing procedures, and type of printers and software [62].

Color stability: Color plays a significant role in choosing the type of material, particularly the materials used in the manufacture of teeth and their denture bases. The lower the mean values of the color change (ΔE_ab_), the higher the color stability of the material. A clinically acceptable ∆E value should not exceed 3.3 [46]. The results of our study showed that the conventional resin had poor color stability at both measurement times. In contrast, the 3D-printed resin demonstrated a lower ΔE_ab_ value of 2.31 ± 1.10, which is below the threshold limit. Previous studies evaluating the color stability of printed resins have favored 3D-printed resins over the conventional heat-cured resins in terms of color property [6,66,67,68]. Alfouzan et al. [6] evaluated the color stability of two 3D-printed resins and one conventional resin in different staining solutions and demonstrated significantly increased color changes in the conventional resin relative to both 3D-printed resins. Conversely, Gruber et al. [5] and Arora et al. [36] demonstrated significantly enhanced discoloration and low color stability in 3D-printed resins compared with milled and heat-cured resins. The authors attributed high water sorption and surface deterioration to the low color stability of the 3D-printed resins. The authors further stated that surface deterioration is inversely correlated with filler concentration, and the majority of 3D-printed resins contain reduced inorganic fillers.

The current study is not without limitations. The findings of this study are contradictory to most of the information in the dental literature, which demonstrates that 3D-printed denture base resins have a performance that is inferior to that of conventional heat-cured PMMA resins. However, these outcomes should be considered carefully because of the variations in the specimen preparation, test set-up, and aging process, and differences in the material compositions of the tested conventional heat-cured and 3D-printed resins. The mechanical tests utilized in this study differ from an actual clinical scenario where cyclical load would have accurately reproduced the masticatory stress. The mechanical and chemical denture hygiene process was not considered in this study; the inclusion of this could have possibly produced a different outcome. In the future, more in vitro research should be performed incorporating different brands of 3D-printed materials and evaluating other properties, such as biocompatibility, precision and accuracy, water sorption and solubility, depth of cure, and reparability, in addition to mechanical and surface properties.

## 5. Conclusions

Within the limitations of the results of this study, the following conclusions can be drawn:

a. The 3D-printed resin had comparable flexural and tensile strength and significantly superior compressive, hardness, and color stability compared with the conventional resin.
b. Aging significantly reduced the flexural strength (−27%), tensile strength (−44%), and hardness (−7%) of the 3D-printed resin in contrast to the conventional resin’s compressive strength (−15%) and color stability
c. The color difference of the 3D-printed resin was below the clinically acceptable value of ΔE_ab_ < 3.3 at both measurement times; the conventional heat-cured resin demonstrated clinically acceptable values of ΔE_ab_ > 3.3.

## Figures and Tables

**Table 1:** Materials used in this study.

Acrylic Resin/Fabrication Methods	Composition	Manufacturer
Unidesa Idobase/ Conventional Flasking	Powder: Polymethyl methacrylate based copolymer >99%, Catalysts and pigments <1% Monomer: Methyl methacrylate >90%, Dimethacrylate <10%	Unidesa-Odi, Madrid, Spain
Formlabs Original Pink (OP)/3D Printing	Methacrylate monomer (40–60%), Urethane dimethacrylate (30–50%), Propylidynetrimethyl trimethacrylate (5–10%), Diphenyl (2,4,6-trimethylbenzoyl) phosphine Oxide (<3%)	Formlabs Inc., Somerville, MA, USA

**Table 2:** Description of the test set-up and specimens.

Tests/Analysis	Method/ISO Specifications	Specimen Shape and Quantity
Flexural strength and elastic modulus	Three-point bending in a universal testing machine. ISO 178:2019 [39]	Rectangular specimens − Conventional: 20 specimens − 3D printed: 20 specimens
Tensile strength	Tensile forces in opposite directions in a universal testing machine. ISO 527-2:2012 [40]	Dumbbell-shaped specimens − Conventional: 20 specimens − 3D printed: 20 specimens
Compressive strength	Compression force applied towards the specimen surface in a universal testing machine. ISO 5883:2002 [41]	Cylindrical specimens − Conventional: 20 specimens − 3D printed: 20 specimens
Hardness	Vickers hardness number in a microhardness tester ISO 6507-1:2023 [42]	Round specimens − Conventional: 6 specimens − 3D printed: 6 specimens
Color stability	Color measurements using a spectrophotometer and CIELab 76-color formula. ISO 28642:2016 [43]	Round specimens − Conventional: 6 specimens − 3D printed: 6 specimens
PMMA material composition	FT-IR spectrophotometer	Round pellets − Conventional: 2 specimens − 3D printed: 2 specimens
For all the above tests, 50% of the specimens were subjected to baseline measurements and the other 50% of the specimens were subjected to thermocycling.		

**Table 3:** CIE Lab color values of two denture base materials before and after thermocycling.

	Baseline		After Thermocycling	
CIE Lab	Conventional	3D-Printed	Conventional	3D-Printed
L*	46.30 ± 1.57	36.65 ± 0.74	50.16 ± 2.49	39.96 ± 4.79
A*	10.1 ± 0.45	4.4 ± 0.16	8.95 ± 0.57	4.73 ± 1.3
B*	1 ± 0.17	−8.38 ± 0.14	0.83 ± 0.14	−4.21 ± 1.78

**Table 4:** IR absorption spectra of the study materials based on reference PMMA.

Wavenumber (cm^−1^)		Functional Group
Conventional	3D-Printed	
2958	2972	Methyl (CH_3_)
1724	1707	Methacrylate (C=O)
2878	2857	Methylene (CH_2_)
1271	1244	Acrylate (C=O-O)
1153	1105	C-O

## Data Availability

The original data presented in the study are included in the article. Further inquiries can be directed to the corresponding author.

## References

[1] Gao Y., Zhang J., Liang J., Yuan D., Zhao W. (2022). Research progress of poly(methyl methacrylate) microspheres: Preparation, functionalization and application. Eur. Polym. J..

[2] Pawar E.G. (2016). A Review Article on Acrylic PMMA. IOSR J. Mech. Civ. Eng..

[3] Hacker M.C., Mikos A.G., Atala A., Lanza R., Thomson J.A., Nerem R. (2011). Chapter 33—Synthetic Polymers. Principles of Regenerative Medicine.

[4] Ali U., Karim K.J.B.A., Buang N.A. (2015). A Review of the Properties and Applications of Poly (Methyl Methacrylate) (PMMA). Polym. Rev..

[5] Gruber S., Kamnoedboon P., Özcan M., Srinivasan M. (2020). CAD/CAM Complete Denture Resins: An In Vitro Evaluation of Color Stability. J. Prosthodont..

[6] Alfouzan A.F., Alotiabi H.M., Labban N., Al-Otaibi H.N., Al Taweel S.M., AlShehri H.A. (2021). Color stability of 3D-printed denture resins: Effect of aging, mechanical brushing and immersion in staining medium. J. Adv. Prosthodont..

[7] Dayan C., Guven M.C., Gencel B., Bural C. (2019). A Comparison of the Color Stability of Conventional and CAD/CAM Polymethyl Methacrylate Denture Base Materials. Acta Stomatol. Croat..

[8] Anadioti E., Musharbash L., Blatz M.B., Papavasiliou G., Kamposiora P. (2020). 3D printed complete removable dental prostheses: A narrative review. BMC Oral Health.

[9] Lee S., Hong S.-J., Paek J., Pae A., Kwon K.-R., Noh K. (2019). Comparing accuracy of denture bases fabricated by injection molding, CAD/CAM milling, and rapid prototyping method. J. Adv. Prosthodont..

[10] Majeed H.F., Hamad T.I., Bairam L.R. (2024). Enhancing 3D-printed denture base resins: A review of material innovations. Sci. Prog..

[11] Fatalla A.A., Tukmachi M.S., Jani G.H. (2020). Assessment of some mechanical properties of PMMA/silica/zirconia nanocomposite as a denture base material. IOP Conf. Ser. Mater. Sci. Eng..

[12] Al-Dwairi Z.N., Tahboub K.Y., Baba N.Z., Goodacre C.J., Özcan M. (2019). A Comparison of the Surface Properties of CAD/CAM and Conventional Polymethylmethacrylate (PMMA). J. Prosthodont..

[13] Alfouzan A.F., AlNouwaisar A.N., AlAzzam N.F., Al-Otaibi H.N., Labban N., Alswaidan M.H., Al Taweel S.M., Alshehri H.A. (2021). Power brushing and chemical denture cleansers induced color changes of pre-polymerized CAD/CAM denture acrylic resins. Mater. Res. Express.

[14] Dimitrova M., Vlahova A., Kalachev Y., Kazakova R., Capodiferro S. (2024). Future Prospects and Challenges in Additive Manufacturing for Complete Dentures: A Narrative Review. Oral.

[15] Schweiger J., Stumbaum J., Edelhoff D., Güth J.F. (2018). Systematics and concepts for the digital production of complete dentures: Risks and opportunities. Int. J. Comput. Dent..

[16] Steinmassl P.-A., Wiedemair V., Huck C., Klaunzer F., Steinmassl O., Grunert I., Dumfahrt H. (2017). Do CAD/CAM dentures really release less monomer than conventional dentures?. Clin. Oral Investig..

[17] Charoenphol K., Peampring C. (2023). Fit Accuracy of Complete Denture Base Fabricated by CAD/CAM Milling and 3D-Printing Methods. Eur. J. Dent..

[18] Anadioti E., Kane B., Soulas E. (2018). Current and Emerging Applications of 3D Printing in Restorative Dentistry. Curr. Oral Health Rep..

[19] Stansbury J.W., Idacavage M.J. (2016). 3D printing with polymers: Challenges among expanding options and opportunities. Dent. Mater..

[20] Alhallak K., Hagi-Pavli E., Nankali A. (2023). A review on clinical use of CAD/CAM and 3D printed dentures. Br. Dent. J..

[21] Javaid M., Haleem A. (2019). Current status and applications of additive manufacturing in dentistry: A literature-based review. J. Oral. Biol. Craniofacial Res..

[22] Cristache C.M., Totu E.E., Iorgulescu G., Pantazi A., Dorobantu D., Nechifor A.C., Isildak I., Burlibasa M., Nechifor G., Enachescu M. (2020). Eighteen Months Follow-Up with Patient-Centered Outcomes Assessment of Complete Dentures Manufactured Using a Hybrid Nanocomposite and Additive CAD/CAM Protocol. J. Clin. Med..

[23] Tasaka A., Matsunaga S., Odaka K., Ishizaki K., Ueda T., Abe S., Yoshinari M., Yamashita S., Sakurai K. (2019). Accuracy and retention of denture base fabricated by heat curing and additive manufacturing. J. Prosthodont. Res..

[24] Raszewski Z., Chojnacka K., Mikulewicz M. (2023). Effects of Surface Preparation Methods on the Color Stability of 3D-Printed Dental Restorations. J. Funct. Biomater..

[25] Kashyap R.U., Nalinakshamma M., Shetty S., Rao S. (2018). Color stability of heat-cured polymethyl methacrylate denture base resin coated with titanium dioxide upon storage in different beverages. J. Interdiscip. Dent..

[26] Bacali C., Badea M., Moldovan M., Sarosi C., Nastase V., Baldea I., Chiorean R.S., Constantiniuc M. (2019). The Influence of Graphene in Improvement of Physico-Mechanical Properties in PMMA Denture Base Resins. Materials.

[27] Chhabra M., Nanditha Kumar M., RaghavendraSwamy K.N., Thippeswamy H.M. (2022). Flexural strength and impact strength of heat-cured acrylic and 3D printed denture base resins—A comparative in vitro study. J. Oral Biol. Craniofacial Res..

[28] Hayran Y., Keskin Y. (2019). Flexural strength of polymethyl methacrylate copolymers as a denture base resin. Dent. Mater. J..

[29] Gad M.M., Fouda S.M., Abualsaud R., Alshahrani F.A., Al-Thobity A.M., Khan S.Q., Akhtar S., Ateeq I.S., Helal M.A., Al-Harbi F.A. (2022). Strength and Surface Properties of a 3D-Printed Denture Base Polymer. J. Prosthodont..

[30] Dimitrova M., Corsalini M., Kazakova R., Vlahova A., Chuchulska B., Barile G., Capodiferro S., Kazakov S. (2022). Comparison between Conventional PMMA and 3D Printed Resins for Denture Bases: A Narrative Review. J. Compos. Sci..

[31] Al-Dwairi Z., Al Haj Ebrahim A., Baba N. (2022). A Comparison of the Surface and Mechanical Properties of 3D Printable Denture-Base Resin Material and Conventional Polymethylmethacrylate (PMMA). J. Prosthodont..

[32] Valenti C., Isabella Federici M., Masciotti F., Marinucci L., Xhimitiku I., Cianetti S., Pagano S. (2024). Mechanical properties of 3D printed prosthetic materials compared with milled and conventional processing: A systematic review and meta-analysis of in vitro studies. J. Prosthet. Dent..

[33] Yu H.-J., Kang Y.-J., Park Y., Kim H., Kim J.-H. (2024). A comparison of the mechanical properties of 3D-printed, milled, and conventional denture base resin materials. Dent. Mater. J..

[34] Fiore A.D., Meneghello R., Brun P., Rosso S., Gattazzo A., Stellini E., Yilmaz B. (2022). Comparison of the flexural and surface properties of milled, 3D-printed, and heat polymerized PMMA resins for denture bases: An in vitro study. J. Prosthodont. Res..

[35] Sonam D., Dayalan M., Fatima S., Sasirekha K. (2021). Comparative evaluation of impact and flexural strength of 3D printed, CAD/CAM milled and heat activated polymethyl methacrylate resins—An in vitro study. Int. J. Sci. Res..

[36] Arora O., Ahmed N., Nallaswamy D., Ganapathy D., Srinivasan M. (2024). Denture base materials: An in vitro evaluation of the mechanical and color properties. J. Dent..

[37] Aguirre B.C., Chen J.H., Kontogiorgos E.D., Murchison D.F., Nagy W.W. (2020). Flexural strength of denture base acrylic resins processed by conventional and CAD-CAM methods. J. Prosthet. Dent..

[38] Alfouzan A.F., Alotiabi H.M., Labban N., Al-Otaibi H.N., Al Taweel S.M., AlShehri H.A. (2021). Effect of aging and mechanical brushing on surface roughness of 3D printed denture resins: A profilometer and scanning electron microscopy analysis. Technol. Health Care Off. J. Eur. Soc. Eng. Med..

[39] (2019). I. Plastics—Determination of Flexural Properties.

[40] (2012). I. Plastics—Determination of Tensile Properties.

[41] (2002). I. Implants for Surgery—Acrylic Resin Cements.

[42] (2023). I. Metallic Materials—Vickers Hardness Test. Part 1: Test Method.

[43] (2016). I.T. Dentistry—Guidance on Colour Measurement.

[44] Colombo M., Poggio C., Lasagna A., Chiesa M., Scribante A. (2019). Vickers Micro-Hardness of New Restorative CAD/CAM Dental Materials: Evaluation and Comparison after Exposure to Acidic Drink. Materials.

[45] Ilie N., Hilton T.J., Heintze S.D., Hickel R., Watts D.C., Silikas N., Stansbury J.W., Cadenaro M., Ferracane J.L. (2017). Academy of Dental Materials guidance-Resin composites: Part I-Mechanical properties. Dent. Mater..

[46] Kotanidis A., Kontonasaki E., Koidis P. (2019). Color alterations of a PMMA resin for fixed interim prostheses reinforced with silica nanoparticles. J. Adv. Prosthodont..

[47] Kavda S., Golfomitsou S., Richardson E. (2023). Effects of selected solvents on PMMA after prolonged exposure: Unilateral NMR and ATR-FTIR investigations. Herit. Sci..

[48] Choksi R.H., Mody P.V. (2016). Flexural properties and impact strength of denture base resins reinforced with micronized glass flakes. J. Indian. Prosthodont. Soc..

[49] Ataei K., Ghaffari T., Moslehifard E., Dizaj S.M. (2024). Physico-chemical and Mechanical Assessments of a New 3D Printed PMMA-Based Acrylic Denture Base Material. Open Dent. J..

[50] Temizci T., Bozoğulları H.N. (2024). Effect of thermal cycling on the flexural strength of 3-D printed, CAD/CAM milled and heat-polymerized denture base materials. BMC Oral Health.

[51] Sai Teja R., Subhabrata M., Dhanraj G. (2022). Effect of Thermocycling Aging on Denture Base Resin—A Comparative Analysis. J. Coast. Life Med..

[52] Alaseef N., Albasarah S., Al Abdulghani H., Al-Harbi F.A., Gad M.M., Akhtar S., Khan S.Q., Ateeq I.S., al-Qarni F.D. (2022). CAD-CAM Fabricated Denture Base Resins: In Vitro Investigation of the Minimum Acceptable Denture Base Thickness. J. Prosthodont..

[53] Al-Qarni F., Gad M. (2022). Printing Accuracy and Flexural Properties of Different 3D-Printed Denture Base Resins. Materials.

[54] Zeidan A.A.E., Abd Elrahim R.A., Abd El Hakim A.F., Harby N.M., Helal M.A. (2022). Evaluation of Surface Properties and Elastic Modulus of CAD-CAM Milled, 3D Printed, and Compression Moulded Denture Base Resins: An In Vitro Study. J. Int. Soc. Prev. Community Dent..

[55] Durkan R., Oyar P. (2018). Comparison of Mechanical and Dynamic Mechanical Behaviors of Different Dental Resins Polymerized by Different Polymerization Techniques. Niger. J. Clin. Pract..

[56] Kwon J.S., Kim J.Y., Mangal U., Seo J.Y., Lee M.J., Jin J., Yu J.H., Choi S.H. (2021). Durable Oral Biofilm Resistance of 3D-Printed Dental Base Polymers Containing Zwitterionic Materials. Int. J. Mol. Sci..

[57] Tian Y., Chen C., Xu X., Wang J., Hou X., Li K., Lu X., Shi H., Lee E.-S., Jiang H.B. (2021). A Review of 3D Printing in Dentistry: Technologies, Affecting Factors, and Applications. Scanning.

[58] Altarazi A., Haider J., Alhotan A., Silikas N., Devlin H. (2022). Assessing the physical and mechanical properties of 3D printed acrylic material for denture base application. Dent. Mater..

[59] Hamdy T.M. (2023). Evaluation of compressive strength, surface microhardness, solubility and antimicrobial effect of glass ionomer dental cement reinforced with silver doped carbon nanotube fillers. BMC Oral Health.

[60] Mohammed H., Ibrahim A.-F., Moaaed M. (2018). Enhancement of the Tensile and the Compression Properties for Heat- Cured Acrylic Resin Denture Base Materials. Baghdad Sci. J..

[61] Alharbi N., Osman R., Wismeijer D. (2016). Effects of build direction on the mechanical properties of 3D-printed complete coverage interim dental restorations. J. Prosthet. Dent..

[62] Lourinho C., Salgado H., Correia A., Fonseca P. (2022). Mechanical Properties of Polymethyl Methacrylate as Denture Base Material: Heat-Polymerized vs. 3D-Printed—Systematic Review and Meta-Analysis of In Vitro Studies. Biomedicines.

[63] Alshali S., Basunbul G., Basunbul A., Giordano Ii R. (2024). Comparison of the flexural strength of printed and milled denture base materials. BMC Oral Health.

[64] Digholkar S., Madhav V.N., Palaskar J. (2016). Evaluation of the flexural strength and microhardness of provisional crown and bridge materials fabricated by different methods. J. Indian Prosthodont. Soc..

[65] Prpić V., Schauperl Z., Ćatić A., Dulčić N., Čimić S. (2020). Comparison of Mechanical Properties of 3D-Printed, CAD/CAM, and Conventional Denture Base Materials. J. Prosthodont..

[66] El Naggar S.M., Helal E., Khalil M.F., Esmat A.M., Elboraey A.N. (2022). Color stability of heat polymerized complete dentures and 3D printed CAD/CAM dentures: A cross-over clinical study. J. Arab Soc. Med. Res..

[67] Khayat A. (2021). The Effect of Denture Cleansers on the Mechanical and Optical Properties of 3D Printed and Heat-Polymerized Dentures.

[68] Raffaini J.C., Soares E.J., Oliveira R.F.d.L., Vivanco R.G., Amorim A.A., Pereira A.L.C., Pires-de-Souza F.C.P. (2023). Effect of artificial aging on mechanical and physical properties of CAD-CAM PMMA resins for occlusal splints. J. Adv. Prosthodont..

